# New Marine Fungal Deoxy-14,15-Dehydroisoaustamide Resensitizes Prostate Cancer Cells to Enzalutamide

**DOI:** 10.3390/md21010054

**Published:** 2023-01-14

**Authors:** Sergey A. Dyshlovoy, Olesya I. Zhuravleva, Jessica Hauschild, Tobias Busenbender, Dmitry N. Pelageev, Anton N. Yurchenko, Yuliya V. Khudyakova, Alexandr S. Antonov, Markus Graefen, Carsten Bokemeyer, Gunhild von Amsberg

**Affiliations:** 1 Laboratory of Experimental Oncology, Department of Oncology, Hematology and Bone Marrow Transplantation with Section Pneumology, Hubertus Wald-Tumorzentrum—University Cancer Center Hamburg (UCCH), University Medical Center Hamburg-Eppendorf, 20246 Hamburg, Germany; 2 Institute of High Technologies and Advanced Materials, Far Eastern Federal University, Vladivostok 690922, Russia; 3 G.B. Elyakov Pacific Institute of Bioorganic Chemistry, Far Eastern Branch of the Russian Academy of Sciences, Prospect 100-Letiya Vladivostoka, Vladivostok 690022, Russia; 4 Martini-Klinik Prostate Cancer Center, University Hospital Hamburg-Eppendorf, 20246 Hamburg, Germany

**Keywords:** *Penicillium dimorphosporum*, secondary metabolites, prenylated indole alkaloid, prostate cancer, androgen receptor, enzalutamide

## Abstract

Marine fungi serve as a valuable source for new bioactive molecules bearing various biological activities. In this study, we report on the isolation of a new indole diketopiperazine alkaloid deoxy-14,15-dehydroisoaustamide (**1**) from the marine-derived fungus *Penicillium dimorphosporum* KMM 4689 associated with a soft coral. The structure of this metabolite, including its absolute configuration, was determined by HR-MS, 1D and 2D NMR as well as CD data. Compound **1** is a very first deoxyisoaustamide alkaloid possessing two double bonds in the proline ring. The isolated compound was noncytotoxic to a panel of human normal and cancer cell lines up to 100 µM. At the same time, compound **1** resensitized prostate cancer 22Rv1 cells to androgen receptor (AR) blocker enzalutamide. The mechanism of this phenomenon was identified as specific drug-induced degradation of androgen receptor transcription variant V7 (AR-V7), which also resulted in general suppression of AR signaling. Our data suggest that the isolated alkaloid is a promising candidate for combinational therapy of castration resistant prostate cancer, including drug-resistant subtypes.

## 1. Introduction

Deoxyisoaustamide alkaloids are the most common derivatives of azocinoindole found in nature [1]. To date, these compounds have only been reported as fungal metabolites. It is highly likely that they are biosynthetically produced and formed from brevianamide E via cyclization of its reversed prenyl group to the nitrogen of the diketopiperazine ring. So far, only approximately ten members of indoldiketopiperazine alkaloids family have been identified [2]. All of them are characterized by great conservatism in structure. The main producer of deoxyisoaustamide alkaloids is the fungus *Penicillium dimorphosporum* KMM 4689, from which deoxyisoaustamides containing a dihydroxylated proline moiety were previously isolated [3].

The data on biological activity of deoxyisoaustamides and related compounds are very limited. Thus, some okaramines isolated from several species of *Penicillium* and *Aspergillus* revealed insecticidal activity [4,5,6,7]. *ent*-Cycloechinulin from *Aspergillus novofumigatus* showed weak antifungal activity against several pathogenic fungi [8]. A neuroprotective activity has been reported for some related deoxyisoaustamide alkaloids [3]. It is important to note that in the activities mentioned above, no precise molecular target or mode of action has been identified. Several reports published to date lack data on cytotoxic activity of these molecules in human cells. In fact, to the best of our knowledge, cytotoxic activity has not been reported for any deoxyisoaustamide-related molecules.

Previously, our group isolated seven new prenylated indole alkaloids belonging to deoxyisoaustamide family from the coral-derived fungus *Penicillium dimorphosporum* KMM 4689 [3]. In the current research, we further investigate metabolites of this fungal strain as a part of our ongoing search for new bioactive compounds. Indeed, we were able to isolate another new diketopiperazine-type alkaloid deoxy-14,15-dehydroisoaustamide (**1**), which contains the rare 6/5/8/6/5 pentacyclic ring system. Here, we report the isolation, structure determination and biological activity of this natural product compound.

## 2. Results and Discussion

### 2.1. Chemistry

**Figure 1:**
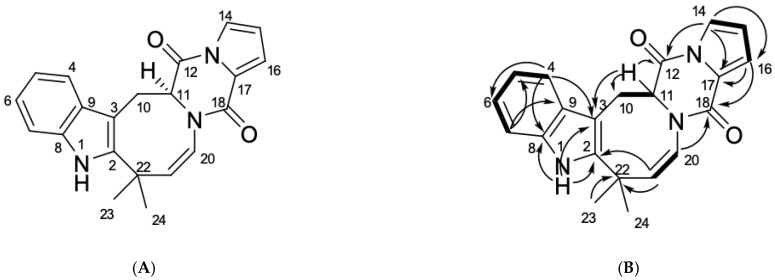
(**A**) Chemical structure and (**B**) key COSY (bold lines) and HMBC (arrows) correlations of the compound **1**.

The fungus was cultured for 21 days on solid rice medium. The EtOAc extract was subjected to repeated column chromatography over silica gel and Gel ODS-A, and then reverse-phase HPLC to yield individual compound 1 (Figure 1).

The molecular formula of 1 was determined as C_21_H_19_N_3_O_2_ from the HRESIMS peak at *m/z* 344.1403 [M − H]− and was in accordance with the ^13^C NMR data.

A close inspection of the ^1^H and ^13^C NMR data of **1** (Table 1; Figures S3–S9) by DEPT and HSQC revealed the presence of an amide proton (δ_H_ 8.05), two methyl groups (δ_H_ 1.40, δ_C_ 32.4, δ_H_ 1.67, δ_C_ 26.4), one sp^3^ methylene (δ_H_ 3.60, 3.78, δ_C_ 28.0), one sp^3^ (δ_H_ 4.52, δ_C_ 60.7), nine olefinic methines (δ_H_ 5.88, δ_C_ 142.6, δ_H_ 5.91, δ_C_ 121.3, δ_H_ 3.07, δ_C_ 115.1, δ_H_ 6.60, δ_C_ 118.7, δ_H_ 6.90, δ_C_ 119.7 δ_H_ 6.96, δ_C_ 121.4, δ_H_ 7.12, δ_C_ 110.2, δ_H_ 7.20, δ_C_ 118.7, δ_H_ 7.31, δ_C_ 117.3), five sp^2^ (δ_C_ 103.0, 124.9, 127.9, 134.2 and 141.0), and one sp^3^ (δ_C_ 37.7) quaternary carbons, as well as two amide carbonyls (δ_C_ 156.0 and 164.7).

The ^1^H–^1^H COSY correlations of H-4/H-5/H-6/H-7 together with the ^1^H-^13^C HMBC correlations from H-1 to C-2 (*δ*_C_ 141.0), C-3 (*δ*_C_ 103.0), C-8 (*δ*_C_ 134.2) and C-9 (*δ*_C_ 127.9), from H-4 to C-3, C-6 (*δ*_C_ 121.4), C-8, C-9 and from H-7 to C-5 (*δ*_C_ 119.7) and C-9 indicated the presence of a disubstituted indole core in **1**. The characteristic NMR data and HMBC correlations H-11 (δ_H_ 4.52)/C-12 (*δ*_C_ 164.7), C-18 (*δ*_C_ 156.0) revealed the presence of diketopiperazine ring in **1**. The correlations observed in the COSY and HSQC spectra of **1** indicated the presence of the following isolated spin systems: >CH–CH_2_– (C-11–C-10), –CH–CH=CH–CH= (C-14–C-16), –CH=CH– (C-20–C-21). The long-range correlations H-14 (δ_H_ 6.60)/C-12, C-16 (δ_C_ 118.7), C-15 (δ_C_ 115.1) and H-16 (δ_H_ 7.20)/C-14 (*δ*_C_ 118.7), C-17 (δ_C_ 124.9) and C-18 indicated that the proline moiety at diketopiperazine ring has two double bonds. The HMBC correlations H_2_-10 (δ_H_ 3.60, 3.78)/C-2, C-3, C-9, C-11 (δ_C_ 60.7), C-12 and H-21 (δ_H_ 5.88)/C-2, C-20 (*δ*_C_ 121.3), C-22 (*δ*_C_ 37.7) and C-24 (*δ*_C_ 32.4) indicated the formation of a closed cycle between the indole and diketopiperazine parts in **1**.

The configuration of the chiral center C-11 in **1** was defined as *S* by the specific rotation that had a positive sign ([α]_D_^20^ + 112.7) just like it has been shown for (+)-deoxyisoaustamide ([α]_D_^20^ +192.0) [9]. Thus, compound **1** was named deoxy-14,15-dehydroisoaustamide. The derivatives containing dehydrogenated proline moiety are rather common in indolediketopiperazine alkaloids (e.g., in brevianamides [10]). At the same time, it should be noted that herein reported deoxy-14,15-dehydroisoaustamide is the very first representative of deoxyisoaustamides which contains dehydrogenated proline.

**Figure 2:**
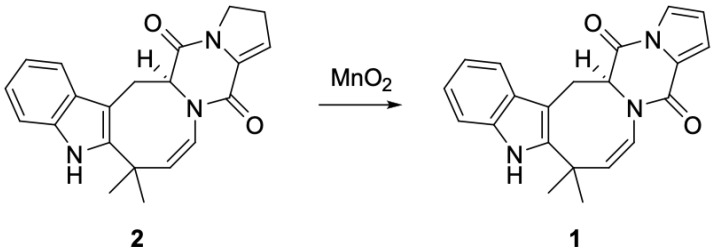
Conversion of (+)-deoxyisoaustamide (**2**) to deoxy-14,15-dehydroisoaustamide (**1**).

In order to confirm the absolute configuration of the isolated deoxy-14,15-dehydroisoaustamide (**1**), we performed a counter synthesis of **1** from the previously isolated (+)-deoxyisoaustamide (**2**) [3]. Thus, refluxing of (+)-deoxyisoaustamide (**2**) in methylene chloride in the presence of active manganese (IV) oxide [11] for 48 h gave deoxy-14,15-dehydroisoaustamide (**1**) with a yield of 30% (Figure 2). All spectral data of the synthesized sample coincided with those for the isolated natural compound **1**. Note this rection reflects a suggested biosynthetic pathway for compound **1**, which is proposed as an enzyme-catalyzed dehydration of (+)-deoxyisoaustamide (**2**).

### 2.2. Biology

#### 2.2.1. Cytotoxicity in Human Prostate Cancer Cells

**Figure 3:**
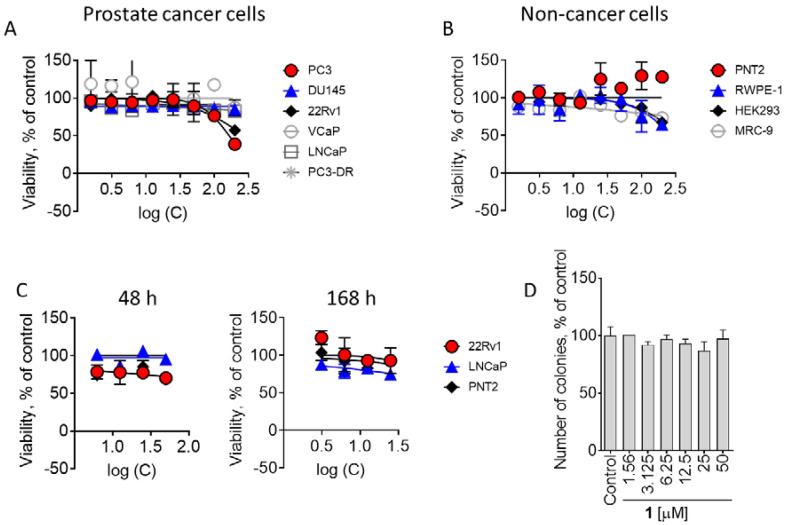
Cytotoxicity of **1** in prostate cancer and non-cancerous cells. (**A**,**B**), Viability of prostate cancer (**A**) and non-cancer (**B**) cell lines measured using a MTT assay after 48 h of treatment. (**C**), Viability of prostate cancer cell lines measured using a trypan blue exclusion assay. Cells were treated with compound **1** for 48 h or 168 h. (**D**), Colony formation assay. 22Rv1 cells were exposed to the indicated concentrations of compound **1** for 48 h; then, the drug-containing media was removed, fresh drug-free medium was added. After additional 14 days of incubation, formed colonies were fixed, stained and counted by naked eye.

To investigate the cytotoxicity of alkaloid **1,** we performed a screening using six human prostate cancer cell lines bearing different levels of resistance to standard therapeutics (Figure 3A). Thus, the following cell lines were included: (i) AR-negative PC3 and DU145 cells harboring resistance to various hormonal and standard chemotherapeutics [12]; (ii) docetaxel-resistant PC3-DR cells derived from PC3 cells by long-term treatment with docetaxel; (iii) AR-FL- (androgen receptor full length) and AR-V7-positive (androgen receptor splice variant V7) 22Rv1 and VCaP cells, which are resistant to hormonal deprivation [12,13]; (iv) AR-FL-positive hormone-dependent LNCaP cells. To determine the selectivity of **1**, cytotoxic effects were additionally examined in four human non-cancerous cells lines (prostate non-cancer PNT2 and RWPE-1 cells, human embryonic kidney HEK 293T cells and human fibroblasts MRC-9 (Figure 3B)). Compound **1** was found to be non-cytotoxic in all the cell lines investigated up to a concentration of 100 µM (Figure 3A,B), even though some cytotoxic effects towards 22Rv1 and PC3 prostate cancer cells were detected at a very high concentration of 200 µM. Additional assays using trypan blue exclusion test did not reveal any pronounced inhibition of cellular proliferation even after a long-term treatment of 168 h (Figure 3C). Finally, a colony formation assay using a pre-plating experimental mode (see Materials and methods) detected no inhibitory activity on colony survival following 14 days of incubation (Figure 3D).

In conclusion, compound **1** was non-cytotoxic to mammalian cells at concentrations of up to 100 µM. In terms of anticancer therapy, lack of cytotoxic activity indicates the molecule to be inactive as a single agent.

#### 2.2.2. Compound **1** Suppresses AR-V7 Expression and Synergizes with Enzalutamide

**Figure 4:**
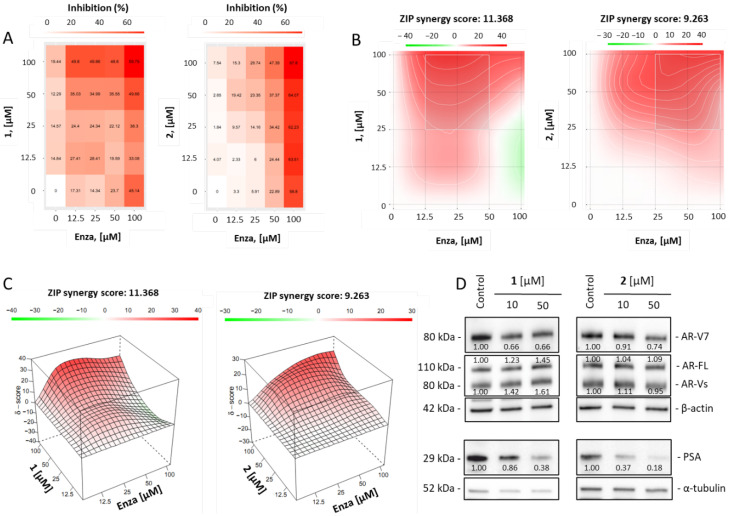
Combinational treatment of 22Rv1 cells with compounds **1** or **2** and enzalutamide (Enza). (**A**–**C**), 22Rv1 cells were treated with **1**, **2**, Enza or combinations **1** + Enza or **2** + Enza for 48 h. Cell viability was determined using MTT assay. (**A**), The heat-map shows the cytotoxic effect (in %) of compound **1**, **2**, Enza and combinations. (**B**,**C**), SynergyFinder 2.0 software and a ZIP reference model were used to calculate and visualize the combinational effect (synergism/additive effect/antagonism) in 2D (**B**) or 3D (**C**). Red regions indicate synergism, white regions refer to additive effect and green regions indicate antagonism. (**D**), Expression of proteins involved in AR-signaling pathway in 22Rv1 cells after 48 h of treatment. The protein expression was measured by Western blotting. β-actin or α-tubulin were used as a loading control. The band intensity was quantified using Image Lab v. 6.1.0. software and normalized to loading control.

As part of our activity screening for new compounds, we then investigated the potential impact of compound **1** on the efficacy of enzalutamide, a clinically approved standard therapy for prostate cancer (Enzo, Figure 4A–D).

Enzalutamide (Enza) is a small molecule capable of inhibiting the androgen receptor (AR). The AR signaling pathway is critical for progression, proliferation and survival of human prostate cancer, especially in the hormone sensitive stage [13]. AR is a transcriptional factor which is activated by androgens. Following androgen binding, AR dimerizes and translocates to the nucleus where it exerts its transcriptional program, leading to cell survival and proliferation. Prostate specific antigen (PSA) is a downstream molecule of the AR signaling pathway, thus increased PSA expression indicates AR to be active. Therefore, PSA expression is widely used to monitor AR activity and thereby prostate cancer progression. The mechanism of AR-signaling suppression exerted by AR targeting agents (ARTAs) may include suppression of androgen production (e.g., by abiraterone) or competitive inhibition of AR (e.g., by enzalutamide). These agents show a high anticancer activity in the hormone sensitive and castration resistant stages of prostate cancer [13]. Unfortunately, over treatment time, prostate cancer cells frequently develop resistance resulting in tumor progression. Alternative splicing of the AR is one of the major mechanisms contributing to AR resistance [14,15]. AR- splice variant 7 (AR-V7) lacks the C-terminal binding domain. Consequently, androgens or antiandrogens (e.g., enzalutamide) lose their ability to interact with AR-V7 [13,14]. However, AR-signaling is not attenuated by the lack of binding partners, as AR-V7 can autoactivate itself despite the absence of androgens. As a result, in AR-V7 expressing cells, the AR-pathway becomes permanently activated, which leads to survival and proliferation of prostate cancer cells [13,14]. 22Rv1 cells express both full length AR (AR-FL) as well as AR-V7 and therefore exert resistance to enzalutamide. In combinational experiments, we found that compound **1** resensitizes drug-resistant 22Rv1 cells to enzalutamide (Figure 4A–C). Hence, we further examined the effects of **1** on AR-V7 expression and observed a dose-dependent down-regulation/degradation of AR-V7 in the treated cells (Figure 4D). Interestingly, the effect on expression of AR-V7 seems to be specific as no down-regulation of wild-type AR-FL or other AR splice variants (AR-Vs) was detected (Figure 4D). At the same time, an effect on total AR signaling was observed by the down-regulation of PSA level (Figure 4D). Importantly, lack of AR-FL degradation allows AR-targeting drugs (e.g., Enza) to bind the receptor, thereby mediating their anticancer activity.

In order to examine whether this biological effect was attributed to the compound **1** exclusively, we tested an effect of compound **2** in combination with Enza using the same experimental models (Figure 4A–D). Notably, compound **2** also induced a selective down-regulation of AR-V7, which expectedly resulted in suppression of PSA expression (Figure 4D) and synergistic effect in combination with Enza (Figure 4B,C). Thus, the above reported biological activity is possessed not only by the deoxy-14,15-dehydroisoaustamide (1) but also by its derivatives. This finding highlights a possibility of further optimization of deoxy-14,15-dehydroisoaustamide structure in order to improve its AR-V7-targeting properties.

Taken together, our data therefore suggest that compound **1** and its derivatives can induce specific down-regulation/degradation of AR-V7, which results in attenuation of resistance mechanism of 22Rv1 cells to Enza and leads to synergistic increase in cytotoxicity of the latter.

## 3. Materials and Methods

### 3.1. General Experimental Procedures

Optical rotations were measured on a Perkin-Elmer 343 polarimeter (Perkin Elmer, Waltham, MA, USA). UV spectra were recorded on a Shimadzu UV-1601PC spectrometer (Shimadzu Corporation, Kyoto, Japan) in methanol. CD spectra were measured with a Chirascan-Plus CD Spectrometer (Leatherhead, UK) in methanol. NMR spectra were recorded in CDCl_3_ on a Bruker Avance III-500 (Bruker BioSpin GmbH, Rheinstetten, Germany) and a Bruker Avance III-700 (Bruker BioSpin GmbH, Rheinstetten, Germany) spectrometers, using TMS as an internal standard. HRESIMS spectra were measured on a Maxis impact mass spectrometer (Bruker Daltonics GmbH, Rheinstetten, Germany). Microscopic examination and photography of fungal cultures were performed with Olympus CX41 microscope fitted with (equipped with) an Olympus SC30 digital camera. Detailed examination of ornamentation of the fungal conidia was performed by scanning electron microscopy (SEM) EVO 40.

Low-pressure liquid column chromatography was performed using Si gel (60/100 μm, Imid Ltd., Krasnodar, Russia) and Gel ODS-A (12 nm, S—75 um, YMC Co., Ishikawa, Japan). Plates precoated with Si gel (5–17 μm, 4.5 × 6.0 cm, Imid Ltd., Krasnodar, Russia) and Si gel 60 RP-18 F_254_S (20 × 20 cm, Merck KGaA, Darmstadt, Germany) were used for thin-layer chromatography. Preparative HPLC was carried out on an Agilent 1100 chromatograph (Agilent Technologies, San Jose, CA, USA) using a YMC ODS-AM (YMC Co., Ishikawa, Japan) (5 µm, 10 × 250 mm) and Kromasil 3-CelluCoat RP (Nouryon, Bohus, Sweden) (5 µm, 4.6 mm × 150 mm) columns with an Agilent 1100 refractometer (Agilent Technologies, USA).

### 3.2. Fungal Strain

The strain P. dimorphosporum KMM 4689 (Collection of Marine Microorganisms, RAS, WDCM #644) was isolated from unidentified soft coral at South China Sea and identified as described earlier [3].

### 3.3. Cultivation of Fungus

The fungus was cultured at 22 °C for three weeks in 60 × 500 mL Erlenmeyer flasks, each containing rice (20.0 g), yeast extract (20.0 mg), KH_2_PO_4_ (10 mg) and natural sea water from the Marine Experimental Station of PIBOC, Troitsa (Trinity) Bay, Sea of Japan (40 mL).

### 3.4. Extraction and Isolation

At the end of the incubation period, the mycelia and medium were homogenized and extracted with EtOAc (1 L). The obtained extract was concentrated to dryness. The residue (1.5 g) was dissolved in H_2_O−EtOH (4:1) (100 mL) and was extracted with *n*-hexane (0.2 L × 3) and EtOAc (0.2 L × 3). After evaporation of the EtOAc layer, the residual material (1.0 g) was passed over a silica column (3 × 14 cm), which was eluted first with *n*-hexane (200 mL) followed by a step gradient from 5% to 50% EtOAc in *n*-hexane (total volume 20 L). Fractions of 250 mL were collected and combined on the basis of TLC (Si gel, toluene–isopropanol 6:1 and 3:1, *v/v*).

The *n*-hexane–EtOAc fraction (70:30, 250 mg) was separated on a Gel ODS-A column (1.5 × 8 cm), which was eluted by a step gradient from 40% to 80% CH_3_OH in H_2_O (total volume 1 L) to yield subfractions A. Subfraction A (60% CH_3_OH, 200 mg) was purified by reverse-phase HPLC on a YMC ODS-AM column eluting with CH_3_CN–H_2_O–TFA (50:50:0.1) and then on a 3-CelluCoat RP column eluting with CH_3_CN–H_2_O (50:50) to yield compound **1** (5.3 mg).

### 3.5. Spectral Data

Deoxy-14,15-dehydroisoaustamide (**1**): amorphous solids; [α]_D_^20^ + 112.7 (*c* 0.11 CH_3_OH); CD (*c* 4.3 × 10^−4^, CH_3_OH), λ_max_(∆ε) 215 (−4.14), 230 (+6.69), 253 (+5.74), 285 (−1.50), 319 (+0.60) nm, see Supplementary Figure S1; UV (CH_3_OH) λmax(log ε) 262 (3.90), 220 (4.38) and 197 (4.32) nm, see Supplementary Figure S2; 1H and 13C NMR data, see Table 1, Supplementary Figures S3–S9; HRESIMS *m/z* 344.1403 [M − H]^−^ (calcd. for C_21_H_18_N_3_O_2_, 344.1405, Δ + 0.6 ppm), 368.1366 [M + Na]^+^ (calcd. for C_21_H_19_N_3_O_2_Na, 368.1369, Δ + 0.9 ppm).

### 3.6. Conversion of (+)-Deoxyisoaustamide (***2***) to Deoxy-14,15-Dehydroisoaustamide (***1***)

Manganese (IV) oxide was added to a solution of 105 mg (0.3 mmol) (+)-deoxyisoaustamide (**2**) in methylene chloride (10 mL). The reaction mixture was refluxed for 48 h. Manganese dioxide was filtered off and washed with methylene chloride. The filtrate was concentrated and subjected to column chromatography (silica gel, CH_2_Cl_2_ → CH_2_Cl_2_-MeOH, 100:1) to give 20 mg of recovered (**1**) and 25 mg (30% based on recovered starting material) of a product identical in all respects with natural deoxy-14,15-dehydroisoaustamide (**1**).

### 3.7. Biological Reagents and Antibodies

The following reagents were used for biological experiments: RNase (Carl Roth, Karlsruhe, Germany); PhosSTOP™ *EASY*pachosphatasease inhibitors cocktail and cOmplete™ *EASY*packs protease inhibitors cocktail (Roche, Mannheim, Germany); MTT (3-(4,5-dimethylthiazol-2-yl)-2,5-diphenyltetrazolium bromide) (Sigma, Taufkirchen, Germany); Enzalutamide (Hycultec GmbH, Beutelsbach, Germany); Tariquidar (MedChemExpress, Monmouth Junction, NJ, USA). Primary and secondary antibodies used are listed in Table 2.

### 3.8. Cell Lines and Culture Conditions

The following human prostate cancer cell lines were purchased from ATCC (Manassas, VA, USA): PC-3, DU145, 22Rv1, and LNCaP, as well as human prostate non-cancer cell line PNT2. MRC-9 (human fibroblast cells) and HEK 293T (human embryonic kidney cells) cell lines were purchased from ECACC (Salisbury, UK). All the cells had a passage number ≤30 at any given time.

The following cell culture conditions were applied: cells were kept as monolayers at 37 °C in a humidified atmosphere with 5% (*v/v*) CO_2_ in the correspondent culture medium: 10% FBS/RPMI medium (RPMI medium supplemented with Glutamax^TM^-I (gibco^®^ Life technologies^TM^, Paisley, UK) containing 10% fetal bovine serum (FBS, gibco^®^ Life technologies^TM^) and 1% penicillin/streptomycin (Invitrogen, Paisley, UK) for PNT2, LNCaP, 22Rv1, PC-3 and DU145 and cells; 10% FBS/DMEM medium (DMEM medium supplemented with Glutamax^TM^-I (gibco^®^ Life technologies^TM^) containing 10% FBS and 1% penicillin/streptomycin (gibco^®^ Life technologies^TM^)) for MRC-9 and HEK 293 cells. Cells were continuously kept in culture for <3 months and were regularly investigated for stable phenotype and mycoplasma infection.

### 3.9. MTT Assay

To evaluate the cytotoxicity of the drugs, a MTT assay was used as previously reported [16]. Cells were preincubated in 96-well plates overnight (6 × 10^3^ cells/well in 100 μL/well). Next, medium was removed and replaced by fresh culture medium (100 μL/well) containing the investigated drugs at the given concentrations. Cells were then treated for 48 h, unless otherwise stated. After treatment, 10 μL/well of 3-(4,5-dimethylthiazol-2-yl)-2,5-diphenyltetrazolium bromide reagent (MTT, 5 mg/mL) were added. Then, cells were additionally incubated for 2–4 h. The medium was removed and the plates were dried. Next, 50 μL of DMSO were added to each well to dissolve the formed crystals and the cell viability was measured using Infinite F200PRO reader (TECAN, Männedorf, Switzerland). GraphPad Prism software v.9.1.1 (GraphPad Software, San Diego, CA, USA) was used to analyze the results. Cells treated with the vehicle alone were used as a control.

### 3.10. Trypan Blue-Based Cell Viability Assay

Trypan blue exclusion assay was performed as described before [16]. In brief, cells (2 × 10^5^ cells/well) were seeded in 6-well plates and incubated overnight. The next day, the medium was replaced with new medium (1 mL/well) containing the tested drugs at indicated concentrations. The cells were treated for 48 h and harvested using trypsination. Next, cells were stained with trypan blue, and the viability was determined automatically using the Beckman Coulter Vi-CELL (Beckman Coulter, Krefeld, Germany). Trypan blue-negative cells were considered as alive cells. GraphPad Prism software v.9.1.1 (GraphPad Software, San Diego, CA, USA) was used to analyze the results. Cells treated with the vehicle alone were used as a control.

### 3.11. Western Blotting

Western Blotting was performed as described before [17]. 10^6^ cells/well in 5 mL/dish were seeded in Petri dishes (ø 6 cm) and incubated overnight. Then, the cells were treated with compounds in fresh culture medium (5 mL/dish) for the indicated time. Next, cells were detached from the bottom via scratching and lysed using a lysis buffer containing protease and phosphatase inhibitors. The protein extracts were separated using electrophoresis in gradient ready-made Mini-PROTEAN^®^ TGX Stain-Free^TM^ gels (Bio-Rad, Hercules, CA, USA). Proteins were then transferred onto ø 0.2 µm pore PVDF membrane. The membrane was blocked using 5% BSA/TBST and consequently incubated with primary and secondary antibodies, and the signals were visualized using ECL chemiluminescence system (Thermo Scientific, Rockford, IL, USA). The used antibodies are listed in Table 2. The band intensity was quantified using Image Lab v. 6.1.0. build 7 software (Bio-Rad Laboratories, Hercules, CA, USA) and normalized to loading control.

### 3.12. Effect of the Drugs in Combinations

In brief, the Zero interaction potency (ZIP) reference model [18] and the SynergyFinder 2.0 software (https://synergyfinder.fimm.fi, accessed on 13 December 2022, [19]) were used to analyze and visualize synergistic, additive or antagonistic effects of the isolated compound in combination with FDA-approved chemotherapeutic drugs. The experiment was performed as previously described [20]. Cells were co-treated with the investigated drugs for indicated time at the indicated concentrations. MTT assay was further used to evaluate the in vitro cytotoxic activity of the individual compounds and their combinations. Synergism is indicated as red areas (positive δ-values), whereas antagonism is indicated as green areas (negative δ-values).

### 3.13. Colony Formation Assay

Cells were seeded in Petri dishes (ø 6 cm, 10^6^ cells in 5 mL/dish) and incubated overnight. The next day, cells were treated for 48 h with different concentrations of the drugs. Following the treatment, cells were trypsinized and the fraction of alive cells was quantified using trypan blue-based exclusion assay (see above). Next, 100 alive cells were seeded into 6-well plates (100 alive cells/well) in drug-free medium (3 mL/well) and incubated for 10 days. Colonies were fixed using 100% methanol (25 min at RT), washed with PBS, air-dried, and stained with Giemsa solution for 25 min at RT. Next, wells were rinsed with dH_2_O and air-dried for 30 min. The number of cell colonies was counted (1 colony was defined as consisting of at least 100 cells).

### 3.14. Data and Atatistical Analysis

All the experiments were executed in triplicates (*n* = 3, biological replicates). Treatment with the vehicle was considered as a control in all experiments. GraphPad Prism v.9.1.1 software (GraphPad Software, San Diego, CA, USA) was used for statistical analyses and calculations of IC_50_s. Data are represented as mean ± standard deviation (SD). For group comparison, the one-way ANOVA followed by Dunnett’s post-hoc tests were used. Statistically significant difference is indicated with asterisk (*), if *p* < 0.05.

## 4. Conclusions

We isolated a new indole diketopiperazine alkaloid deoxy-14,15-dehydroisoaustamide from the extract of a soft coral-associated marine-derived fungus *Penicillium dimorphosporum* KMM 4689 (**1**). Its structure and absolute configuration were determined using HR-MS, NMR, CD and counter synthesis. To the best of our knowledge, compound **1** is the very first deoxyisoaustamide alkaloid possessing two double bonds in the proline ring. At noncytotoxic concentrations compound **1** specifically degraded AR-V7 and therefore could resensitize the cells to AR-inhibitor enzalutamide. Our data indicate that the isolated alkaloid and its derivatives are capable of potentizing the efficacy of AR-targeting agents in treatment resistant prostate cancer, suggesting further preclinical and clinical development.

## Figures and Tables

**Table 1:** ^1^H (*δ* in ppm, *J* in Hz) and ^13^C NMR data (*δ* in ppm) for compound **1**.

Position	1 ^a^		
	^13^C (*δ*C)	^1^H (*δ*H, *J* in Hz)	HMBC
1		8.05 s	2, 3, 8, 9
2	141.0, C		
3	103.0, C		
4	117.3, CH	7.31 d (7.9)	3, 6, 7, 8, 9
5	119.7, CH	6.90 t (7.7)	4, 6, 7, 8, 9
6	121.4, CH	6.96 t (7.7)	4, 5, 7, 8, 9
7	110.2, CH	7.12 d (7.9)	4, 5, 9
8	134.2, C		
9	127.9, C		
10	28.0, CH_2_	α: 3.78 d (14.7) β: 3.60 dd (7.2, 14.7)	2, 3, 9, 11, 12 2, 3, 9, 11, 12
11	60.7, CH	4.52 d (7.3)	3, 10, 12, 18, 20
12	164.7, C		
14	118.7, CH	6.60 dd (1.6, 3.4)	12, 15, 16, 17, 18
15	115.1, CH	6.07 t (3.3)	12, 14, 16, 17, 18
16	118.7, CH	7.20 dd (1.6, 3.3)	14, 15, 17, 18
17	124.9, C		
18	156.0, C		
20	121.3, CH	5.91 d (8.6)	2, 11, 21, 22, 23, 24
21	142.6, CH	5.88 d (8.6)	2, 11, 20, 22, 23, 24
22	37.7, C		
23	26.4, CH_3_	1.67 s	2, 21,22, 23
24	32.4, CH_3_	1.40 s	2, 21, 22, 24

^a^ Chemical shifts were measured at 700.13 and 176.04 MHz in CDCl_3._

**Table 2:** List of antibodies used.

Antibodies	Clonality	Source	Cat.-No.	Dilution	Manufacturer
anti-β-Actin-HRP	pAb	goat	sc-1616	1:10,000	Santa Cruz
anti-α-Tubulin	mAb	mouse	T5168	1:5000	Sigma-Aldrich
anti-AR	pAb	rabbit	sc-816	1:200	Santa Cruz
anti-AR-V7	mAb	rabbit	198394	1:1000	abcam
anti-PSA	mAb	rabbit	#5365	1:1000	Cell Signaling
anti-mouse IgG-HRP		sheep	NXA931	1:10,000	GE Healthcare
anti-rabbit IgG-HRP		goat	#7074	1:5000	Cell Signaling

## Data Availability

The original data are available from the correspondent author on request.

## Supplementary Materials

The following supporting information can be downloaded at: https://www.mdpi.com/article/10.3390/md21010054/s1, Figures S1–S2: CD and UV spectra of compound **1**; Figures S3–S10: 1D and 2D NMR spectra of compound **1**; Figure S11: HRESIMS of compound **1**; Figure S12: HPLC chromatogram of **1**
