# Supplementary material for: New Marine Fungal Deoxy-14,15-Dehydroisoaustamide Resensitizes Prostate Cancer Cells to Enzalutamide
Source: Mar Drugs. 2023 Jan 14;21(1):54. doi: 10.3390/md21010054 (PMC9861654; doi:10.3390/md21010054)
Supplement: Supplementary file 1 [file marinedrugs-21-00054-s001.zip › marinedrugs-2122547-supplementary.pdf]

SUPPLEMENTARY INFORMATION

New Marine Fungal Deoxy-14,15-Dehydroisoaustamide Resensitizes Prostate Cancer Cells to Enzalutamide

Sergey A. Dyshlovoy [1,2,*,†], Olesya I. Zhuravleva [2,3,†], Jessica Hauschild [1], Tobias Busenbender [1], Dmitry N. Pelageev [3], Anton N. Yurchenko [3], Yuliya V. Khudyakova [3], Alexandr S. Antonov [3], Markus Graefen [4], Carsten Bokemeyer [1] and Gunhild von Amsberg [1,4]

1 Laboratory of Experimental Oncology, Department of Oncology, Hematology and Bone Marrow Transplantation with Section Pneumology, Hubertus Wald-Tumorzentrum—University Cancer Center Hamburg (UCCH), University Medical Center Hamburg-Eppendorf, 20246 Hamburg, Germany

[2] Institute of High Technologies and Advanced Materials, Far Eastern Federal University, Vladivostok 690922, Russia
[3] G.B. Elyakov Pacific Institute of Bioorganic Chemistry, Far Eastern Branch of the Russian Academy of Sciences, Prospect 100-Letiya Vladivostoka, Vladivostok 690022, Russia
[4] Martini-Klinik Prostate Cancer Center, University Hospital Hamburg-Eppendorf, 20246 Hamburg, Germany
* Correspondence: s.dyshlovoy@uke.de
† These authors contributed equally to this work.

Content

Figure S1. CD spectrum of **1** 4
Figure S2. UV spectrum of **1** 4
Figure S3. ^{1}H NMR spectrum (700 MHz, $CDCl_3$) of **1** 5
Figure S4. ^{13}C NMR spectrum (176 MHz, $CDCl_3$) of **1** 8
Figure S5. DEPT-135 spectrum (176 MHz, $CDCl_3$) of **1** 10
Figure S6. COSY-45 spectrum (700 MHz, $CDCl_3$) of **1** 11
Figure S7. HSQC spectrum (500 MHz, $CDCl_3$) of **1** 12
Figure S8. HMBC spectrum (500 MHz, $CDCl_3$) of **1** 13
Figure S9. NOESY spectrum (700 MHz, $CDCl_3$) of **1** 14
Figure S10. ^{1}H NMR spectrum (500 MHz, $CDCl_3$) of **1** (synthetic) 15
Figure S11. HR ESI mass spectrum of **1** 17
Figure S12. HPLC chromatogram of **1**. 3-CelluCoat RP (Kromasil, Sweden) (5 µm, 4.6 mm × 150 mm) column, acetonitrile–water (50:50) 18

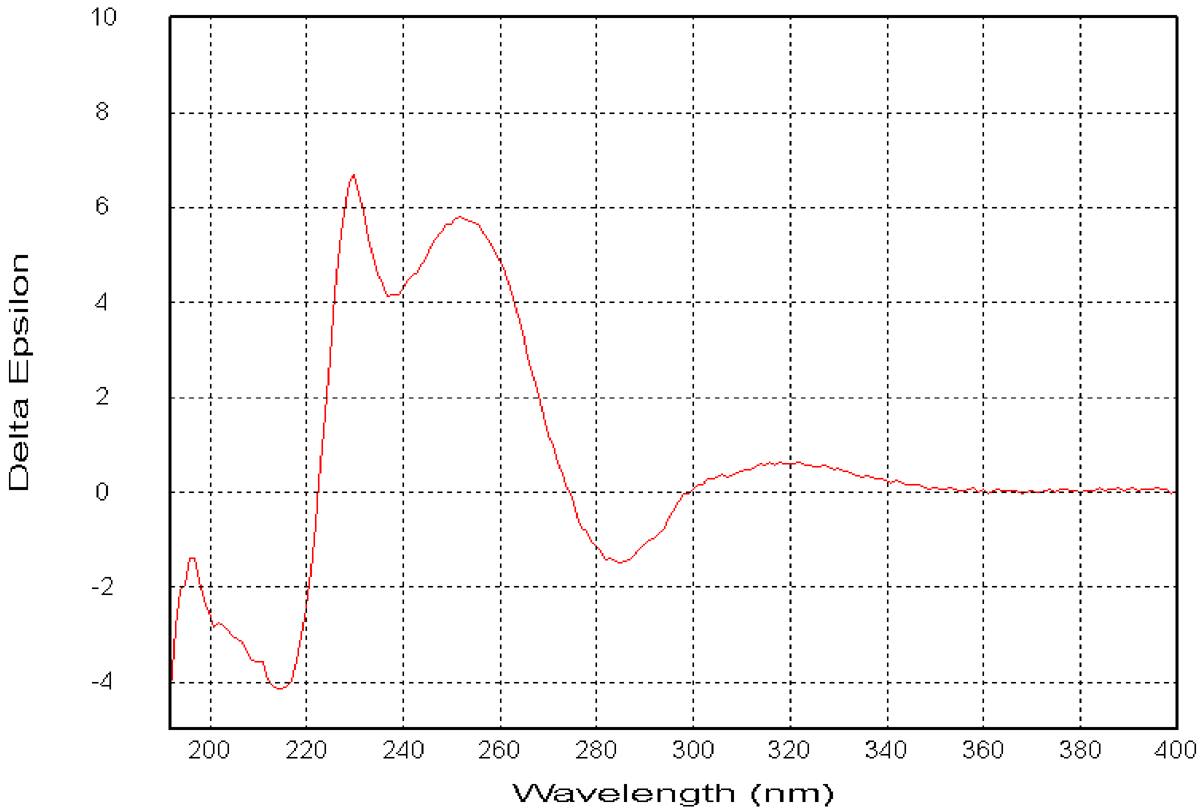

Figure S1. CD spectrum of **1**

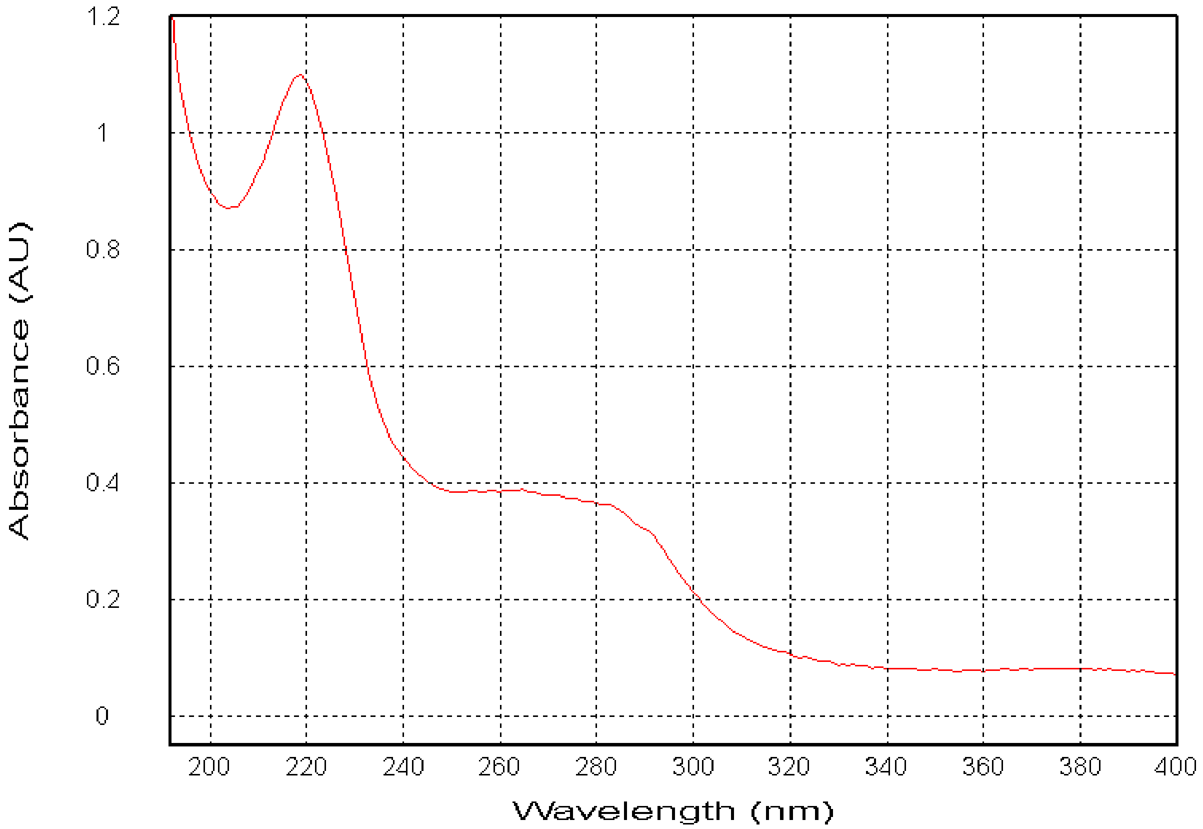

Figure S2. UV spectrum of **1**

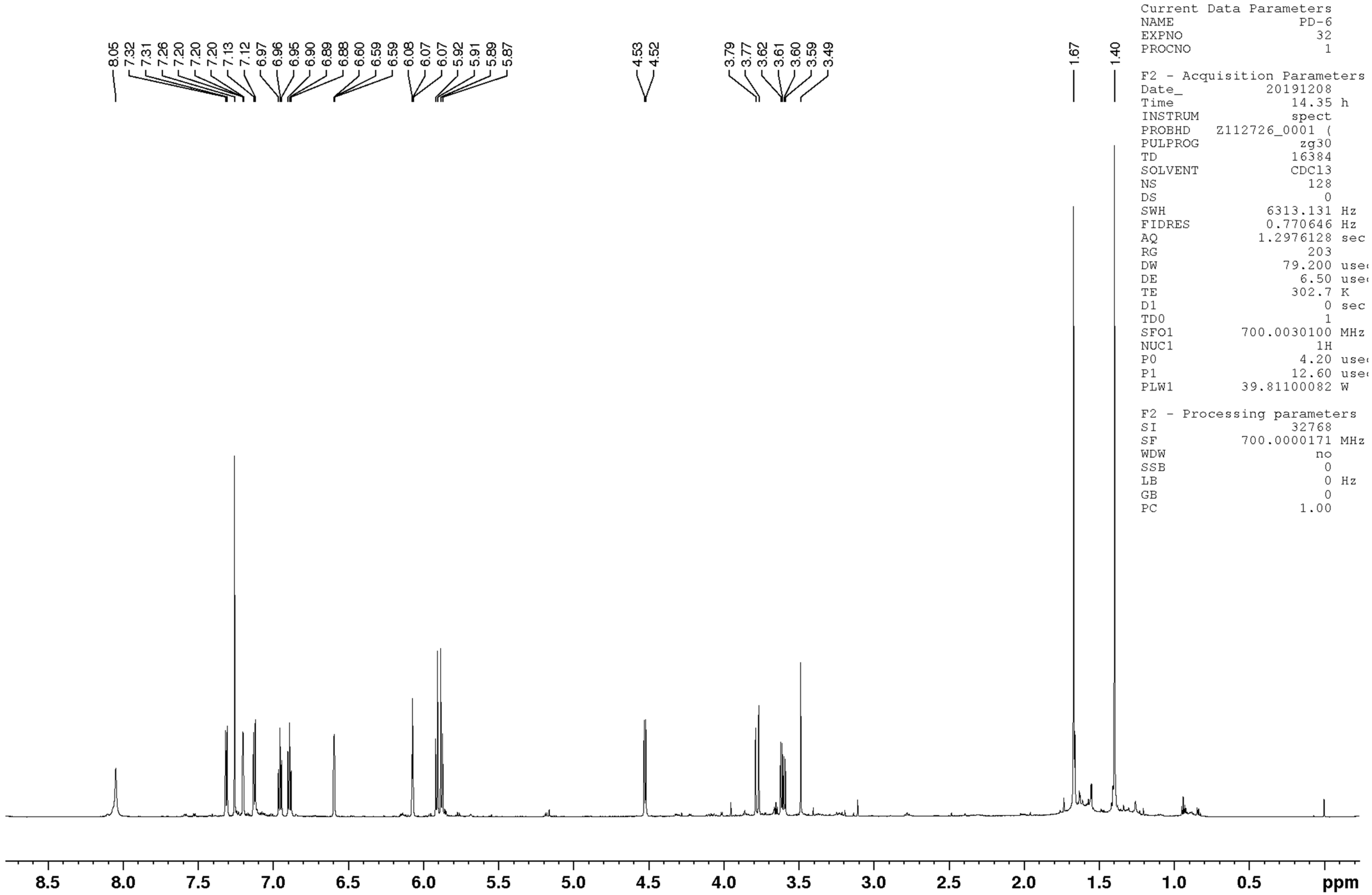

Figure S3. ^{1}H NMR spectrum (700 MHz, $CDCl_3$) of **1**

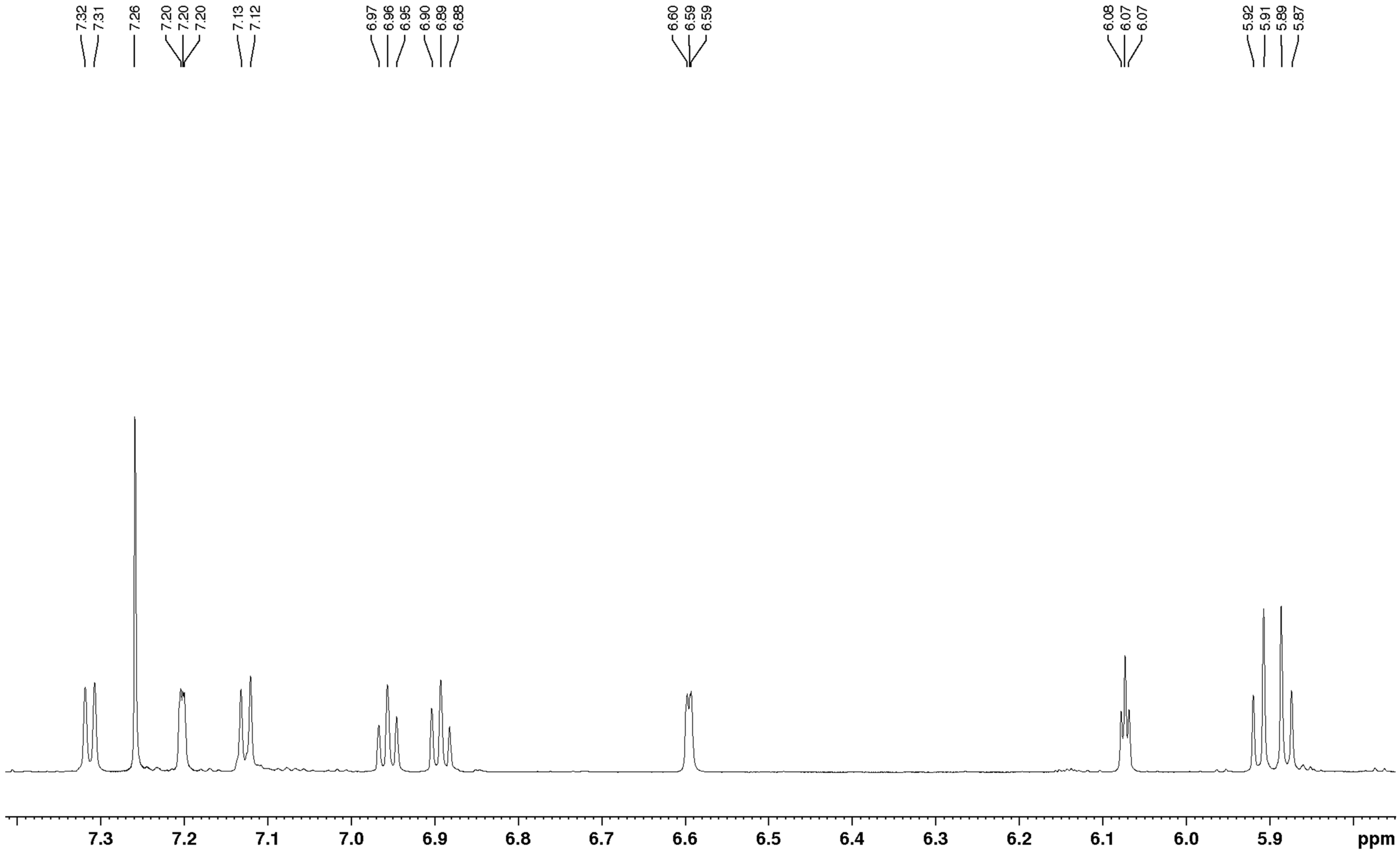

Expanded ^{1}H NMR spectrum of **1**

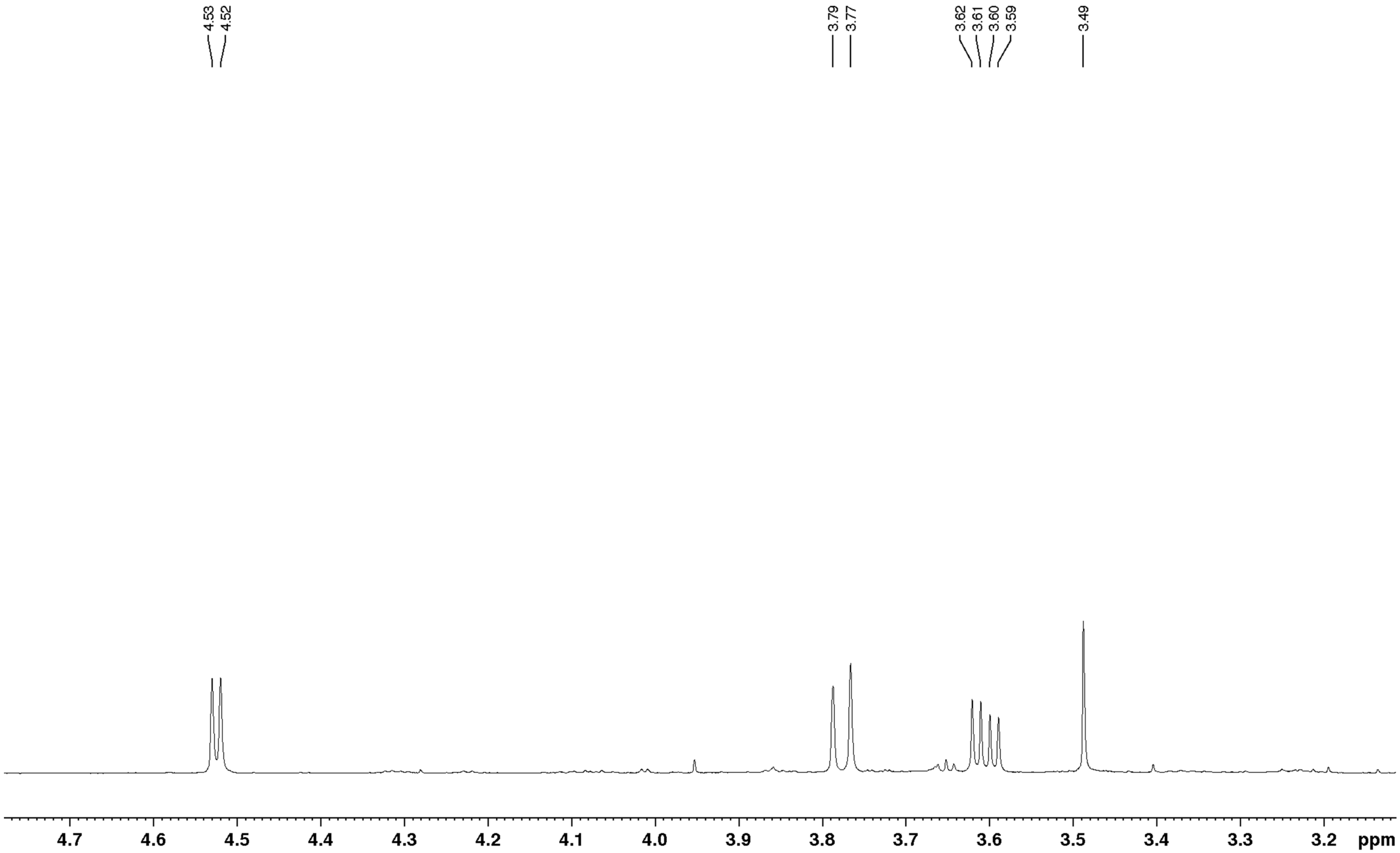

Expanded ^{1}H NMR spectrum of **1**

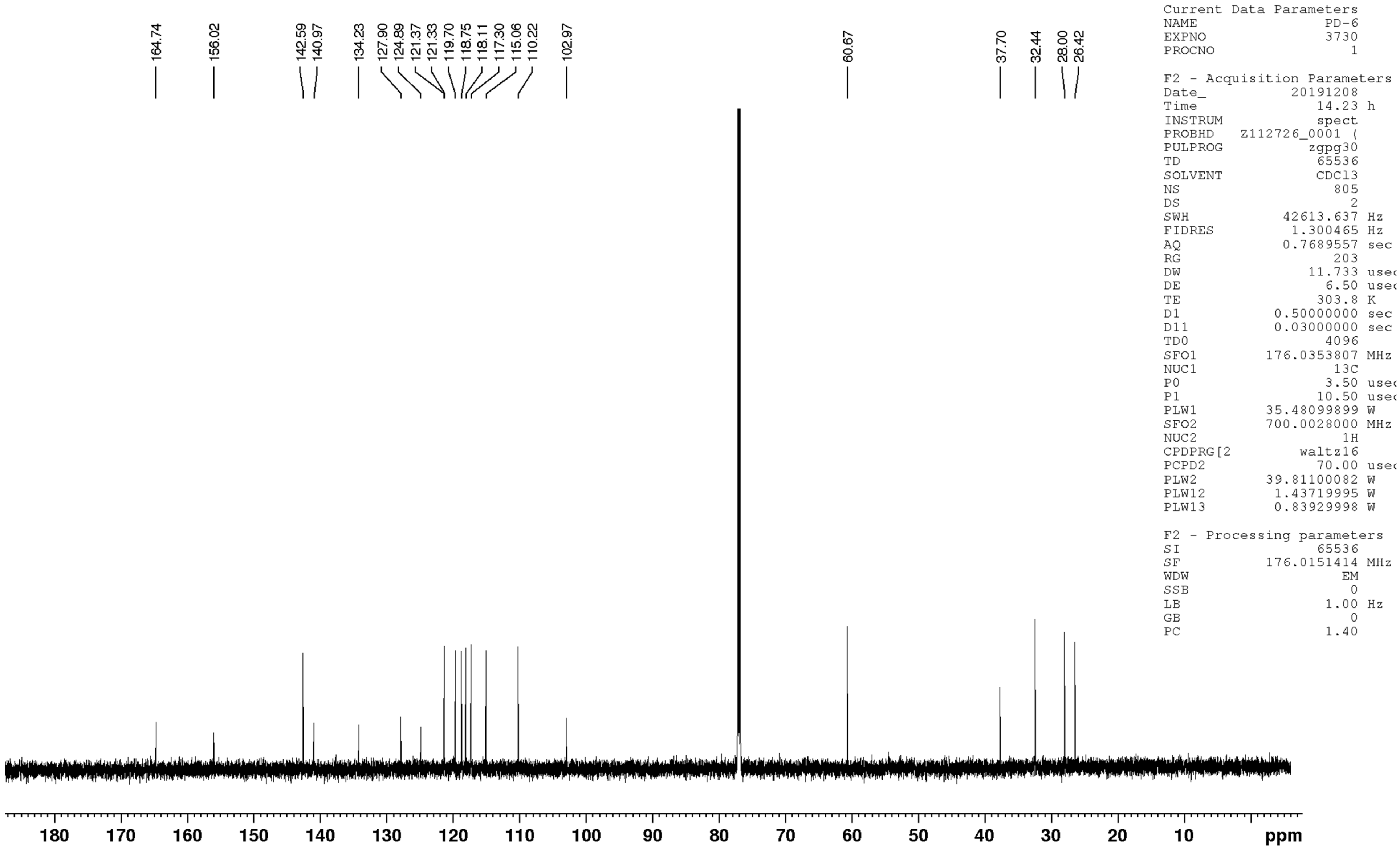

Figure S4. ^{13}C NMR spectrum (176 MHz, $CDCl_3$) of **1**

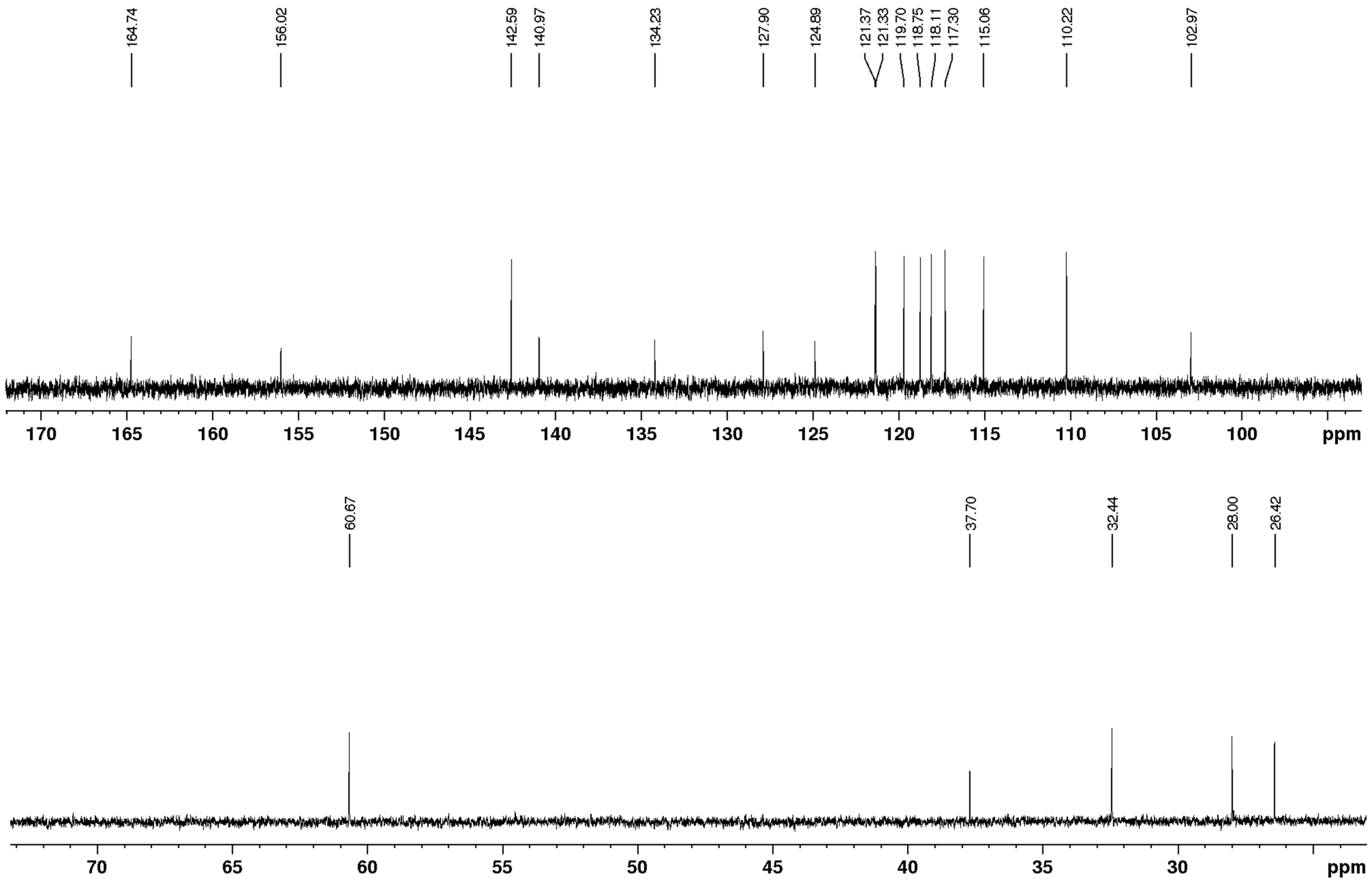

Expanded ^{13}C NMR spectrum of **1**

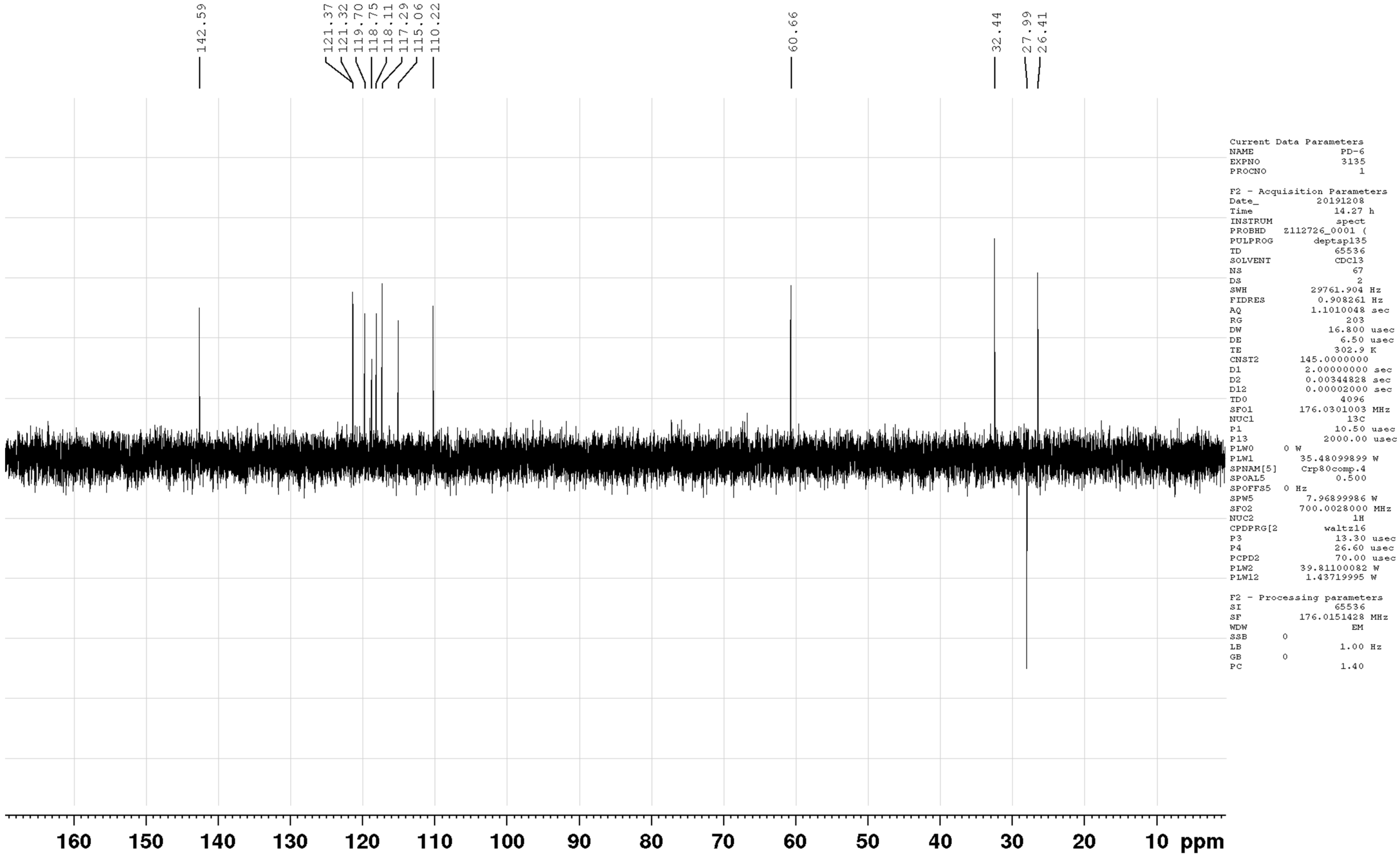

Figure S5. DEPT-135 spectrum (176 MHz, $CDCl_3$) of **1**

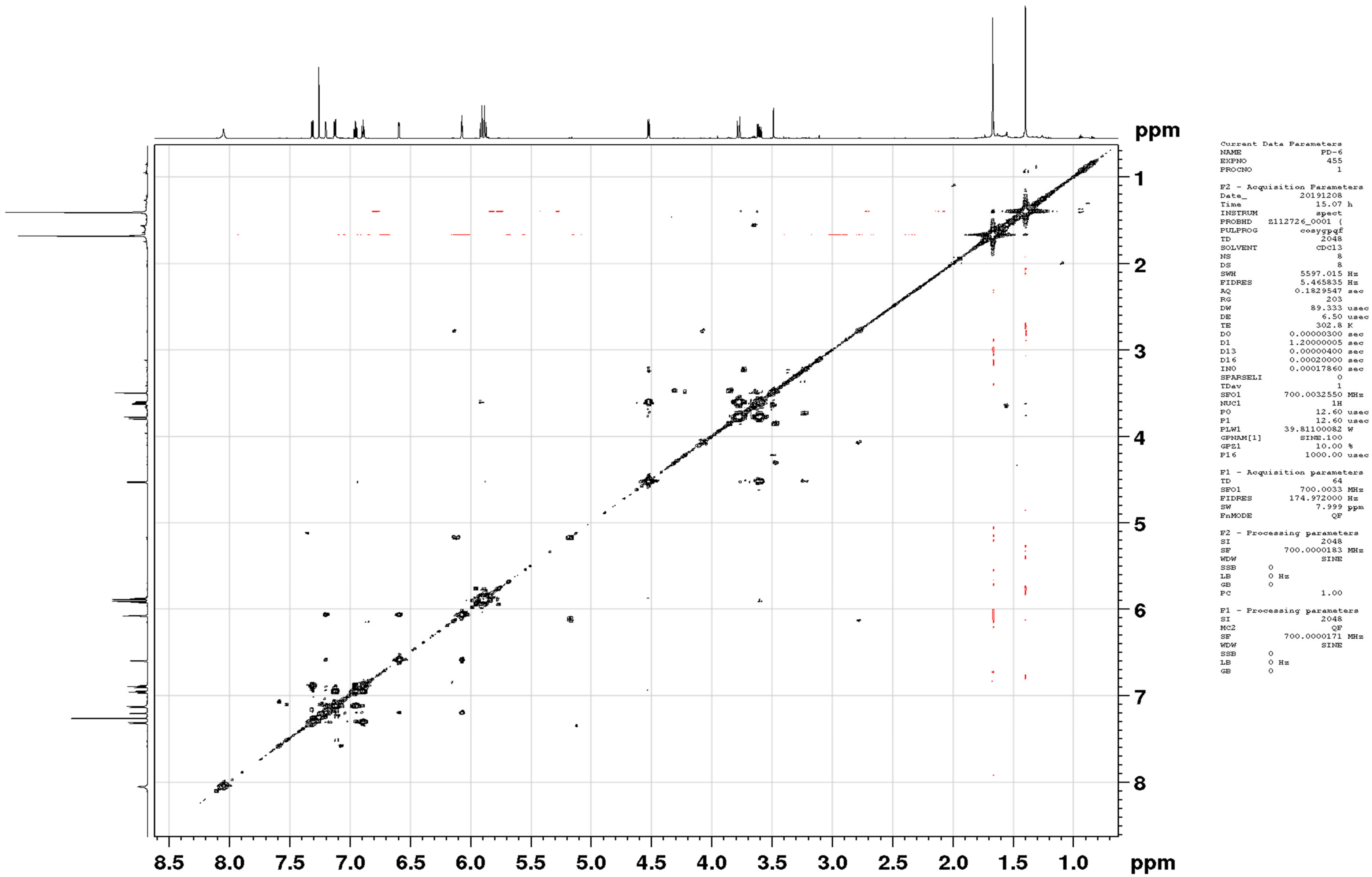

Figure S6. COSY-45 spectrum (700 MHz, $CDCl_3$) of **1**

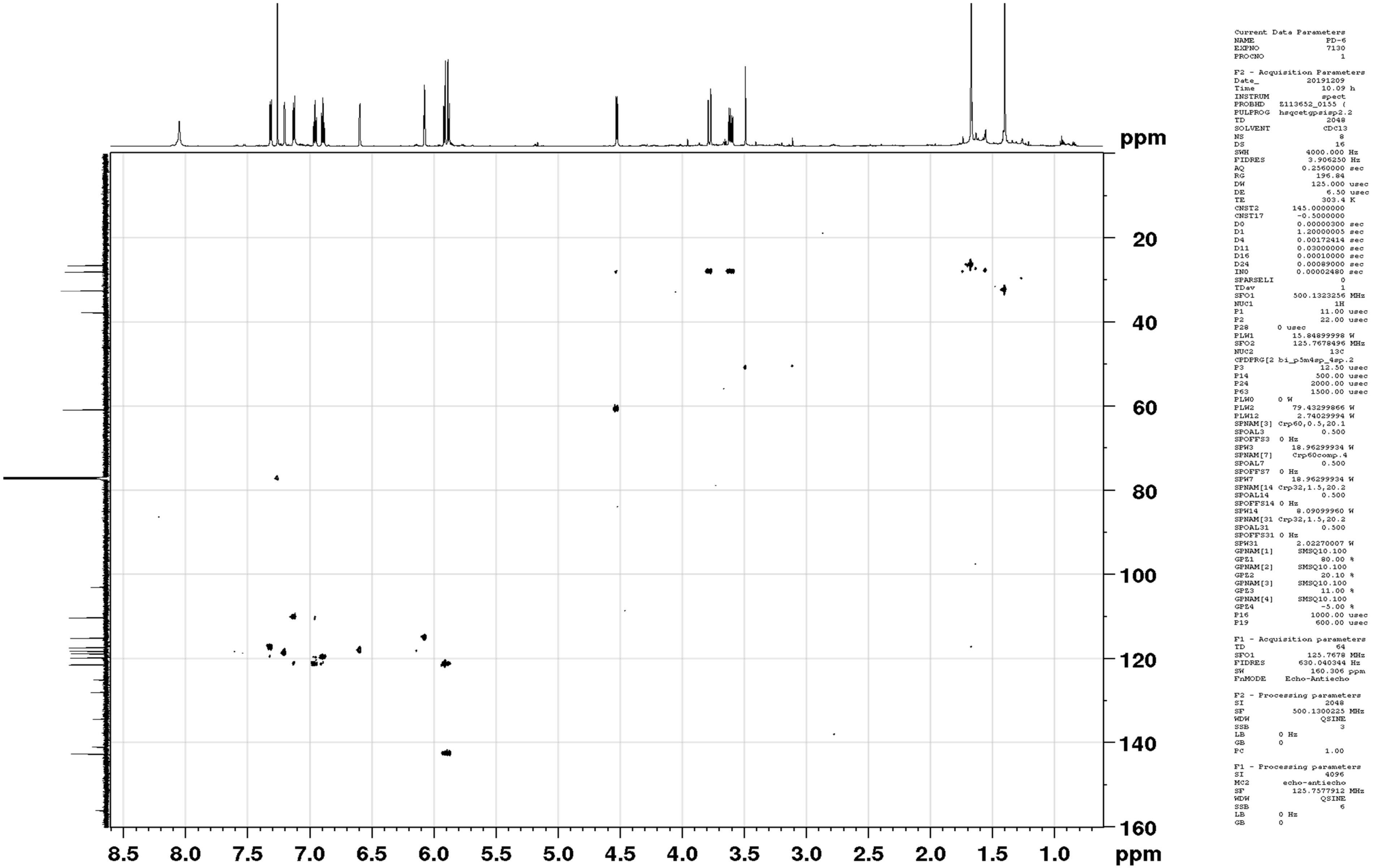

Figure S7. HSQC spectrum (500 MHz, $CDCl_3$) of **1**

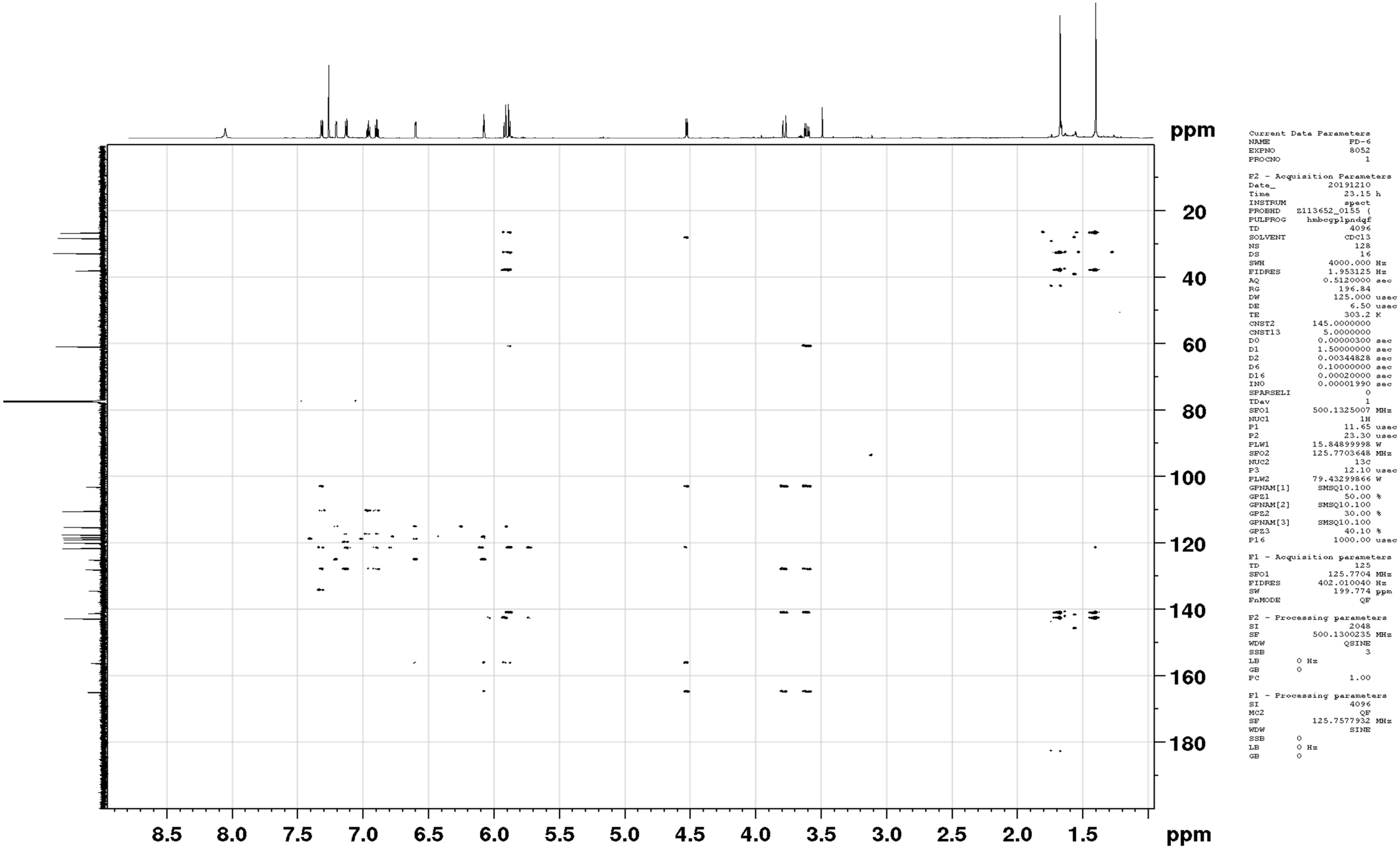

Figure S8. HMBC spectrum (500 MHz, $CDCl_3$) of **1**

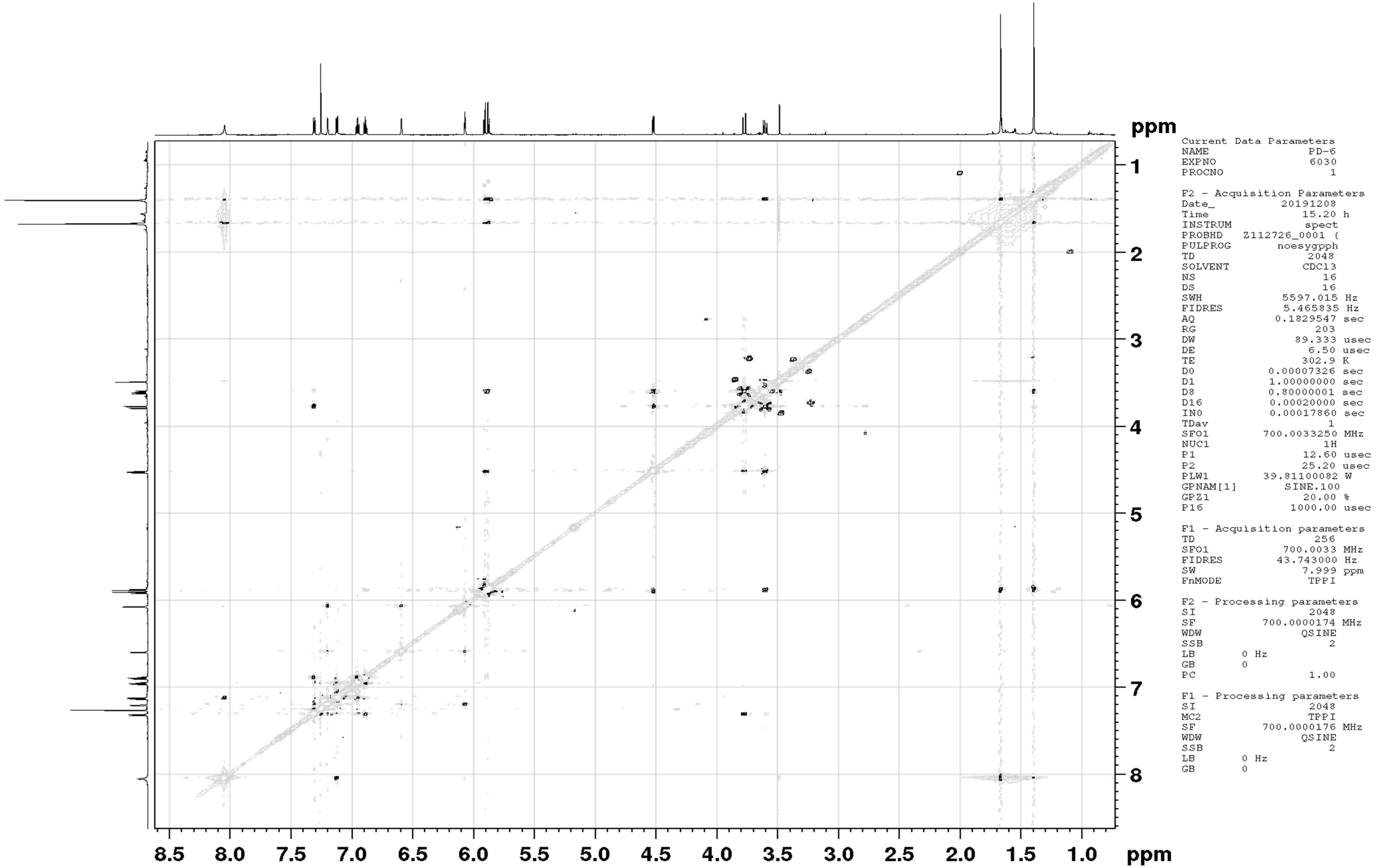

Figure S9. NOESY spectrum (700 MHz, $CDCl_3$) of **1**

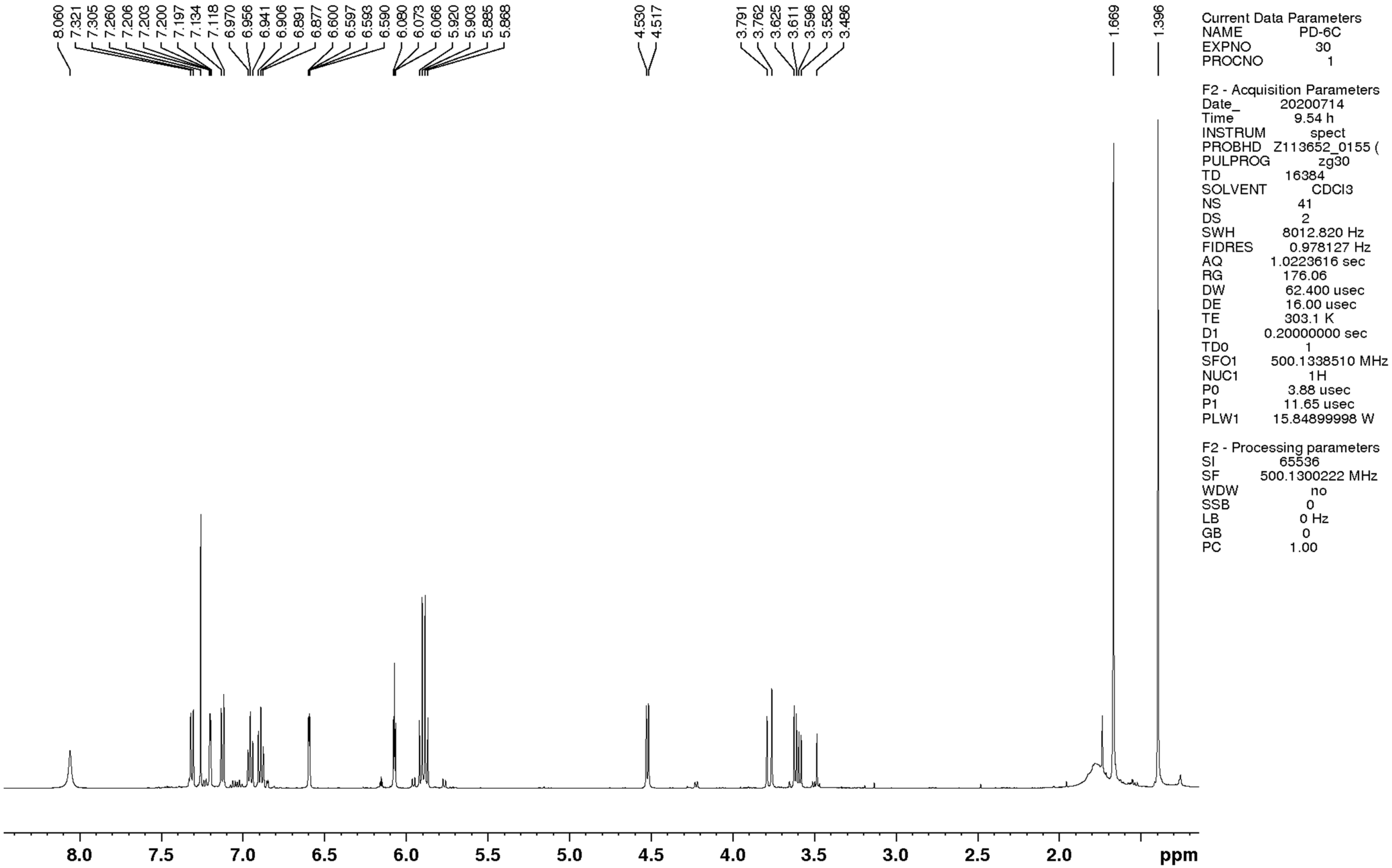

Figure S10. ^{1}H NMR spectrum (500 MHz, $CDCl_3$) of **1** (synthetic)

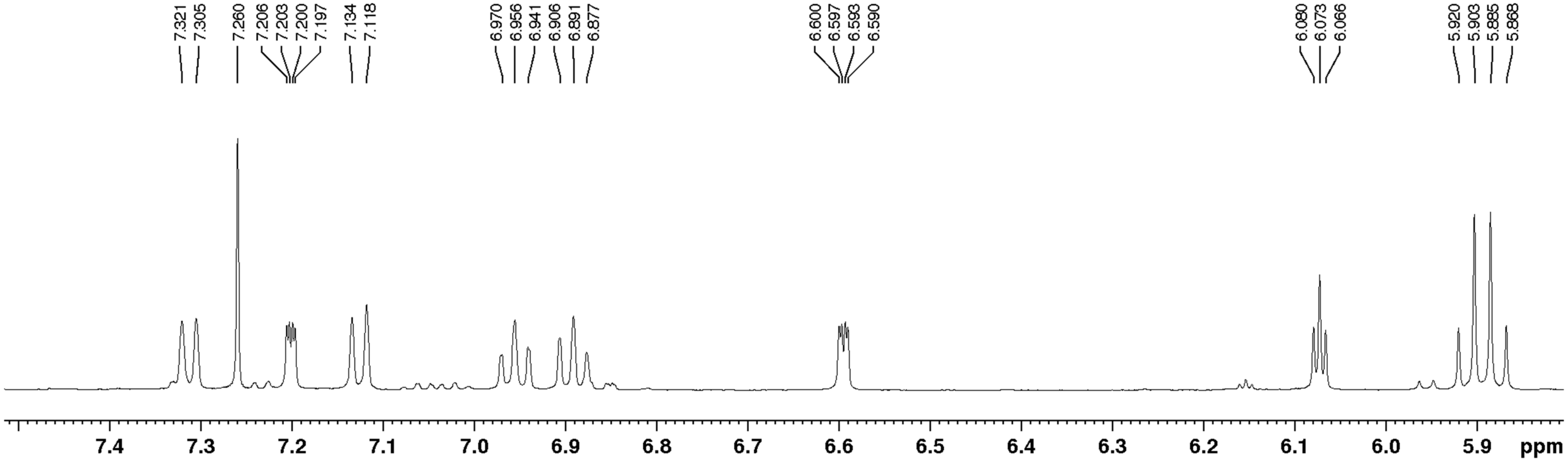

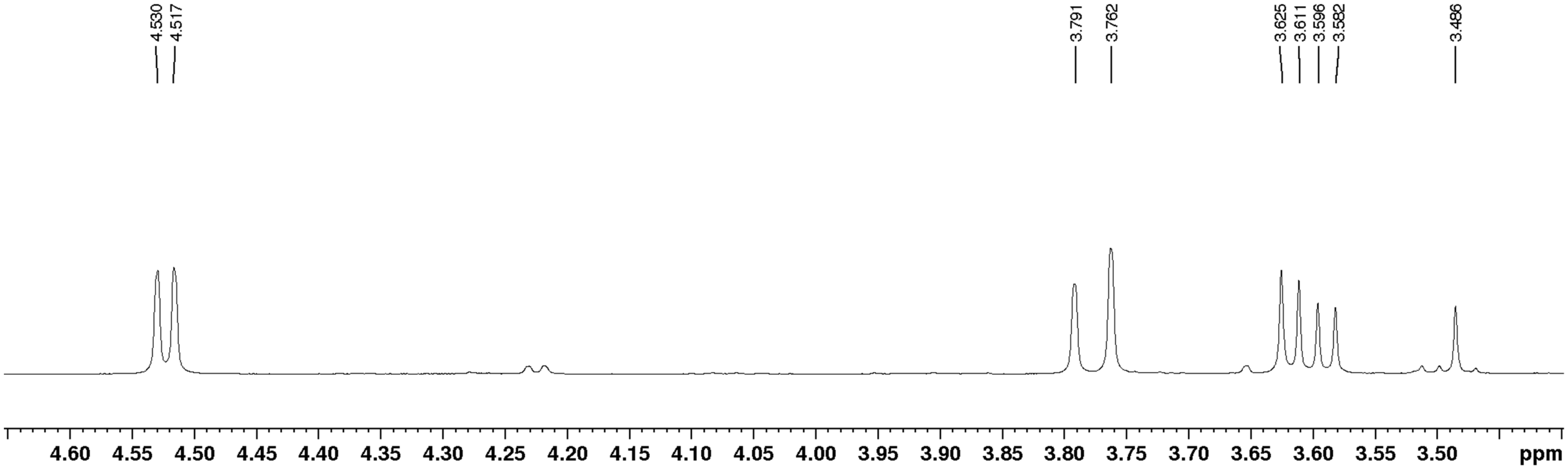

Expanded ^{1}H NMR spectrum of **1** (synthetic)

HR(−)ESI MS

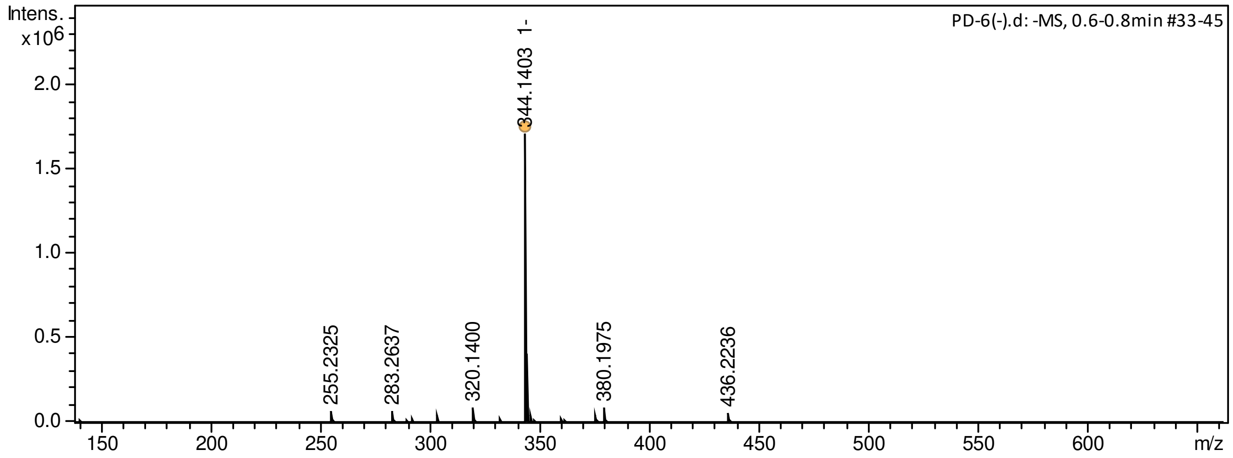

MS/MS

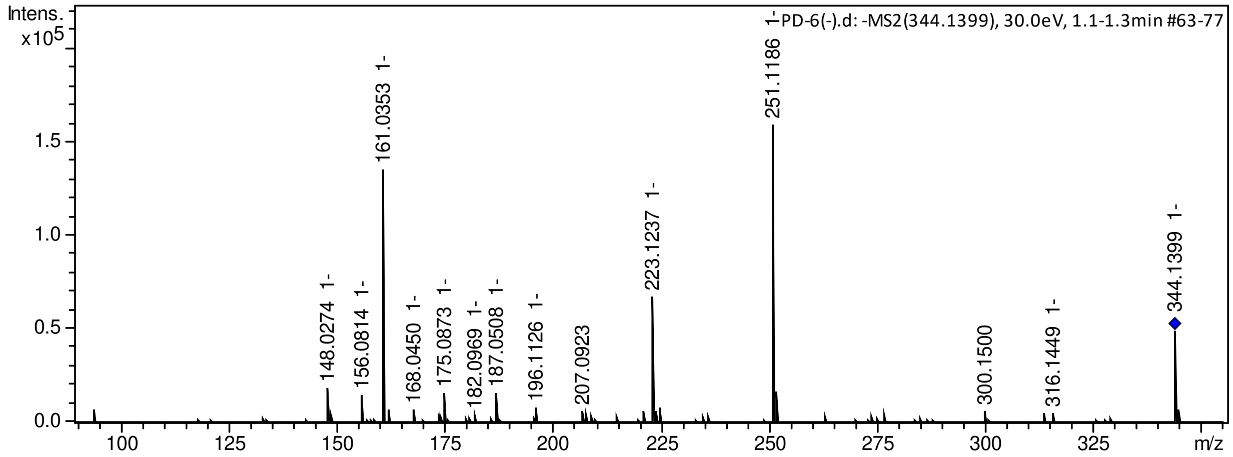

Figure S11. HR ESI mass spectrum of **1**

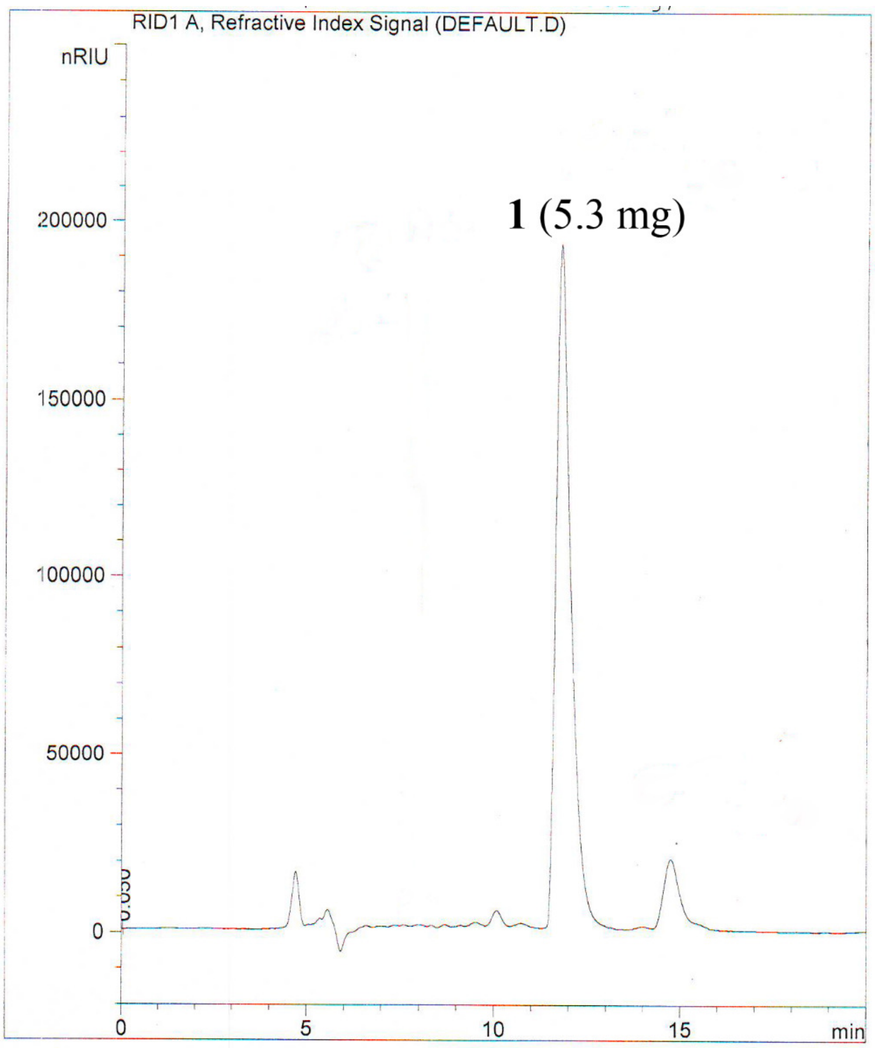

Figure S12. HPLC chromatogram of **1**. 3-CelluCoat RP (Kromasil, Sweden) (5 µm, 4.6 mm × 150 mm) column, acetonitrile–water (50:50)
